# Bis[4-(4-chloro­benzo­yl)-3-methyl-1-phenyl-1*H*-pyrazol-5-olato-κ^2^ *O*,*O*′]bis­(methanol-κ*O*)nickel(II)

**DOI:** 10.1107/S1600536810038973

**Published:** 2010-10-09

**Authors:** Xin Zhang, Meng Huang, Cong Du, Junjing Han

**Affiliations:** a College of Chemistry, Tianjin Normal University, Tianjin 300387, People’s Republic of China

## Abstract

The mol­ecular structure of the neutral mononuclear title complex, [Ni(C_17_H_12_ClN_2_O_2_)_2_(CH_3_OH)_2_], is centrosymmetric. The Ni^II^ atom, which is located on an inversion center, is in a distorted octahedral coordination, defined by four O atoms from two ligands as well as two O atoms from two methanol mol­ecules. Inter­molecular O—H⋯N hydrogen bonds between the hy­droxy group of methanol and a pyrazole N atom link the mol­ecules, forming a two-dimensional network parallel to (100).

## Related literature

For general background to Schiff base compounds in coordin­ation chemistry, see: Harrop *et al.* (2003 ▶); Yu *et al.* (1993 ▶); Wu *et al.* (1993 ▶). For the anti­bacterial properties of Schiff bases derived from 4-acyl-5-pyrazolo­nes and their metal complexes, see: Li *et al.* (1997 ▶, 2004 ▶).
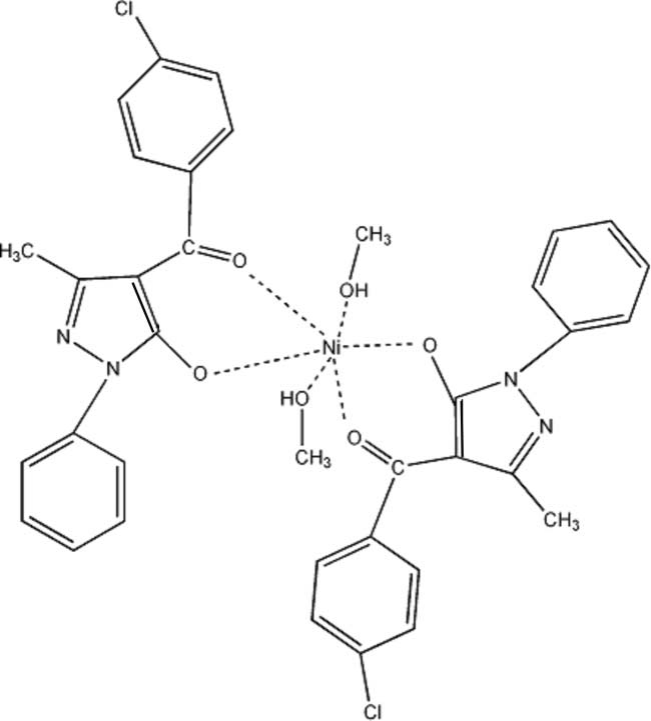


## Experimental

### 

#### Crystal data

[Ni(C_17_H_12_ClN_2_O_2_)_2_(CH_4_O)_2_]
*M* *_r_* = 746.27
Monoclinic, 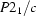

*a* = 11.8398 (7) Å
*b* = 12.3162 (7) Å
*c* = 13.2104 (8) Å
β = 114.706 (1)°
*V* = 1750.03 (18) Å^3^
*Z* = 2
Mo *K*α radiation
μ = 0.76 mm^−1^
*T* = 296 K
0.24 × 0.22 × 0.18 mm

#### Data collection

Bruker SMART CCD area-detector diffractometer
Absorption correction: multi-scan (*SADABS*; Bruker, 1999 ▶) *T*_min_ = 0.834, *T*_max_ = 0.872
8808 measured reflections
3089 independent reflections
2534 reflections with *I* > 2σ(*I*)
*R*_int_ = 0.021

#### Refinement

*R*[*F* ^2^ > 2σ(*F* ^2^)] = 0.034
*wR*(*F* ^2^) = 0.094
*S* = 1.03
3089 reflections
225 parameters
H-atom parameters constrained
Δρ_max_ = 0.37 e Å^−3^
Δρ_min_ = −0.34 e Å^−3^

### 

Data collection: *SMART* (Bruker, 1999 ▶); cell refinement: *SAINT* (Bruker, 1999 ▶); data reduction: *SAINT*; program(s) used to solve structure: *SHELXS97* (Sheldrick, 2008 ▶); program(s) used to refine structure: *SHELXL97* (Sheldrick, 2008 ▶); molecular graphics: *SHELXTL* (Sheldrick, 2008 ▶); software used to prepare material for publication: *SHELXTL*.

## Supplementary Material

Crystal structure: contains datablocks I, global. DOI: 10.1107/S1600536810038973/bh2312sup1.cif

Structure factors: contains datablocks I. DOI: 10.1107/S1600536810038973/bh2312Isup2.hkl

Additional supplementary materials:  crystallographic information; 3D view; checkCIF report

## Figures and Tables

**Table 1:** Hydrogen-bond geometry (Å, °)

*D*—H⋯*A*	*D*—H	H⋯*A*	*D*⋯*A*	*D*—H⋯*A*
O3—H3*A*⋯N2^i^	0.85	2.00	2.795 (2)	156

Symmetry code: (i) 
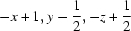
.

**Fig. 1.**
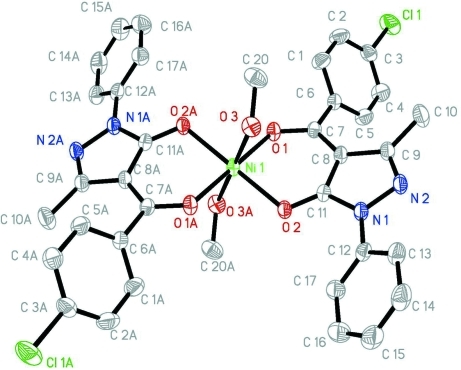
The molecular structure of (I), with anisotropic displacement ellipsoids drawn at the 30° probability level. [Symmetry code:(i)1-x,-y,-z.]

**Fig. 2.**
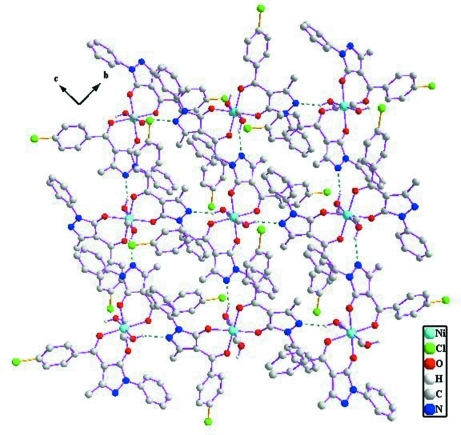
The two-dimensional supra-molecular network of (I) produced by the inter-molecular O-H···N weak hydrogen-bonding interactions.

## supplementary crystallographic information

### Comment

In recent years, Schiff base complexes with metals have generated a wide interest because they possess a large spectrum of biological, pharmaceutical and catalytic properties, such as antitumor and antioxidative activities, as well as the inhibition of lipid peroxidation, among others (Harrop *et al.*, 2003; Yu *et al.*, 1993; Wu *et al.*, 1993). The Schiff bases derived from 4-acyl-5-pyrazolones and their metal complexes have also been widely studied for their high antibacterial activity (Li *et al.*, 1997, 2004). In this paper, we report the synthesis and crystal structure of the title compound, (I), containing a β-ketoamine ligand with organic chlorine, based on a pyrazolone derivative.

The molecular structure of (I) reveals a neutral centrosymmetric mononuclear complex, with the asymmetric unit comprising a half molecule (Fig. 1). The distorted octahedral Ni^II^ center, which locates on a crystallographic inversion center, is coordinated to four O donors from a couple of ligands, and two O atoms from two methanol molecules. The equatorial Ni—O bond lengths are comparable with an average value of 2.0345 (6) Å, which are significantly shorter than that of the axial Ni—O distance of 2.0651 (16) Å. The *cis* bond angles around the Ni^II^ center range from 88.47 (7) to 91.53 (7)°. Intermolecular hydrogen bonds (Table 1) link the molecules together, forming a two-dimensional network (Fig. 2).

### Experimental

A mixture of Ni(OAc)_2_.4H_2_O (24.8 mg, 0.10 mmol), 4(*Z*)-4-((4-chlorophenyl)(hydroxy)methylene)-3-methyl-1-phenyl-1*H*-pyrazol-5(4*H*)-one (62.6 mg, 0.20 mmol) and methanol (12 ml) was heated at 433 K for 2 days in a sealed Teflon-lined stainless steel vessel (20 mL) under autogenous pressure. After the reaction system was slowly cooled down to the room temperature, it was placed to stand at room temperature for a period of four weeks, affording green block crystals in 60% yield.

### Refinement

Although all H atoms were visible in difference maps, they were placed in geometrically calculated positions, with C—H distances in the range 0.93–0.96 Å, and O—H = 0.85 Å. Isotropic displacement parameters were calculated as *U*_iso_(H)=1.2 or 1.5*U*_eq_(carrier atom).

### Crystal data

**Table:**

[Ni(C_17_H_12_ClN_2_O_2_)_2_(CH_4_O)_2_]	*F*(000) = 772
*M**_r_* = 746.27	*D*_x_ = 1.416 Mg m^−^^3^
Monoclinic, *P*2_1_/*c*	Mo *K*α radiation, λ = 0.71073 Å
Hall symbol: -P 2ybc	Cell parameters from 3041 reflections
*a* = 11.8398 (7) Å	θ = 2.4–25.4°
*b* = 12.3162 (7) Å	µ = 0.76 mm^−^^1^
*c* = 13.2104 (8) Å	*T* = 296 K
β = 114.706 (1)°	Block, green
*V* = 1750.03 (18) Å^3^	0.24 × 0.22 × 0.18 mm
*Z* = 2	

### Data collection

**Table:**

Bruker SMART CCD area-detector diffractometer	3089 independent reflections
Radiation source: fine-focus sealed tube	2534 reflections with *I* > 2σ(*I*)
graphite	*R*_int_ = 0.021
φ and ω scans	θ_max_ = 25.0°, θ_min_ = 1.9°
Absorption correction: multi-scan (*SADABS*; Bruker, 1999)	*h* = −14→8
*T*_min_ = 0.834, *T*_max_ = 0.872	*k* = −14→14
8808 measured reflections	*l* = −15→15

### Refinement

**Table:**

Refinement on *F*^2^	Primary atom site location: structure-invariant direct methods
Least-squares matrix: full	Secondary atom site location: difference Fourier map
*R*[*F*^2^ > 2σ(*F*^2^)] = 0.034	Hydrogen site location: inferred from neighbouring sites
*wR*(*F*^2^) = 0.094	H-atom parameters constrained
*S* = 1.03	*w* = 1/[σ^2^(*F*_o_^2^) + (0.0466*P*)^2^ + 0.7427*P*] where *P* = (*F*_o_^2^ + 2*F*_c_^2^)/3
3089 reflections	(Δ/σ)_max_ < 0.001
225 parameters	Δρ_max_ = 0.37 e Å^−^^3^
0 restraints	Δρ_min_ = −0.34 e Å^−^^3^
0 constraints	

### Fractional atomic coordinates and isotropic or equivalent isotropic displacement parameters (Å^2^)

**Table:**

	*x*	*y*	*z*	*U*_iso_*/*U*_eq_	
Ni1	0.5000	0.0000	0.0000	0.03539 (14)	
Cl1	0.04928 (8)	0.55881 (7)	−0.33203 (7)	0.0790 (3)	
O1	0.39759 (15)	0.13217 (12)	−0.07861 (12)	0.0421 (4)	
O2	0.61693 (14)	0.09579 (12)	0.12531 (12)	0.0423 (4)	
O3	0.39208 (16)	−0.01739 (13)	0.08784 (14)	0.0476 (4)	
H3A	0.3970	−0.0784	0.1196	0.071*	
C1	0.1856 (2)	0.2801 (2)	−0.1767 (2)	0.0613 (7)	
H1	0.1567	0.2100	−0.1765	0.074*	
C2	0.1060 (3)	0.3578 (2)	−0.2451 (3)	0.0667 (8)	
H2	0.0234	0.3406	−0.2893	0.080*	
C3	0.1491 (3)	0.4595 (2)	−0.2474 (2)	0.0529 (6)	
C4	0.2709 (3)	0.4855 (2)	−0.1845 (2)	0.0609 (8)	
H4	0.3006	0.5545	−0.1887	0.073*	
C5	0.3491 (2)	0.4082 (2)	−0.1151 (2)	0.0553 (7)	
H5	0.4317	0.4258	−0.0716	0.066*	
C6	0.3072 (2)	0.30524 (18)	−0.10879 (18)	0.0407 (5)	
C7	0.3925 (2)	0.22122 (17)	−0.03395 (18)	0.0379 (5)	
C8	0.4653 (2)	0.24382 (18)	0.08010 (18)	0.0403 (5)	
C9	0.4556 (2)	0.32600 (19)	0.1533 (2)	0.0457 (6)	
N2	0.54567 (19)	0.31717 (16)	0.25322 (17)	0.0483 (5)	
N1	0.61951 (18)	0.22984 (15)	0.24962 (15)	0.0427 (5)	
C11	0.5709 (2)	0.17991 (17)	0.14723 (18)	0.0376 (5)	
C10	0.3568 (3)	0.4093 (2)	0.1350 (2)	0.0698 (9)	
H10A	0.3632	0.4354	0.2056	0.105*	
H10B	0.2764	0.3771	0.0947	0.105*	
H10C	0.3674	0.4687	0.0928	0.105*	
C12	0.7225 (2)	0.19863 (18)	0.34771 (19)	0.0429 (6)	
C13	0.7201 (3)	0.2167 (2)	0.4506 (2)	0.0538 (7)	
H13	0.6501	0.2468	0.4548	0.065*	
C14	0.8222 (3)	0.1898 (2)	0.5459 (2)	0.0695 (9)	
H14	0.8211	0.2023	0.6149	0.083*	
C15	0.9255 (3)	0.1449 (3)	0.5412 (2)	0.0766 (10)	
H15	0.9942	0.1273	0.6063	0.092*	
C16	0.9266 (3)	0.1260 (3)	0.4383 (3)	0.0714 (9)	
H16	0.9963	0.0949	0.4345	0.086*	
C17	0.8256 (2)	0.1529 (2)	0.3416 (2)	0.0543 (7)	
H17	0.8270	0.1402	0.2727	0.065*	
C20	0.2607 (3)	−0.0031 (2)	0.0339 (3)	0.0652 (8)	
H20A	0.2394	0.0676	0.0512	0.098*	
H20B	0.2207	−0.0576	0.0595	0.098*	
H20C	0.2335	−0.0097	−0.0452	0.098*	

### Atomic displacement parameters (Å^2^)

**Table:**

	*U*^11^	*U*^22^	*U*^33^	*U*^12^	*U*^13^	*U*^23^
Ni1	0.0489 (3)	0.0260 (2)	0.0311 (2)	0.00240 (17)	0.01650 (18)	−0.00093 (16)
Cl1	0.0751 (5)	0.0799 (6)	0.0691 (5)	0.0358 (4)	0.0176 (4)	0.0205 (4)
O1	0.0575 (10)	0.0310 (8)	0.0355 (8)	0.0043 (7)	0.0170 (7)	−0.0009 (7)
O2	0.0486 (9)	0.0340 (8)	0.0383 (9)	0.0065 (7)	0.0124 (7)	−0.0067 (7)
O3	0.0580 (11)	0.0439 (10)	0.0459 (10)	0.0027 (8)	0.0266 (8)	0.0103 (7)
C1	0.0505 (16)	0.0468 (16)	0.0721 (19)	−0.0082 (12)	0.0114 (14)	0.0034 (13)
C2	0.0428 (15)	0.067 (2)	0.0699 (19)	−0.0032 (13)	0.0034 (14)	0.0038 (15)
C3	0.0528 (16)	0.0539 (16)	0.0483 (15)	0.0150 (13)	0.0173 (13)	0.0065 (12)
C4	0.0630 (18)	0.0404 (15)	0.0701 (19)	0.0015 (12)	0.0187 (15)	0.0158 (13)
C5	0.0474 (15)	0.0422 (14)	0.0627 (17)	−0.0024 (12)	0.0096 (13)	0.0075 (12)
C6	0.0470 (14)	0.0345 (12)	0.0400 (13)	0.0002 (10)	0.0175 (11)	−0.0025 (10)
C7	0.0440 (13)	0.0309 (12)	0.0397 (12)	−0.0017 (9)	0.0186 (10)	0.0005 (9)
C8	0.0500 (14)	0.0307 (12)	0.0375 (12)	0.0033 (10)	0.0155 (11)	−0.0027 (10)
C9	0.0525 (15)	0.0374 (13)	0.0437 (13)	0.0047 (11)	0.0165 (12)	−0.0054 (11)
N2	0.0574 (13)	0.0392 (11)	0.0458 (12)	0.0072 (9)	0.0191 (10)	−0.0108 (9)
N1	0.0508 (12)	0.0353 (10)	0.0380 (10)	0.0050 (9)	0.0147 (9)	−0.0063 (8)
C11	0.0464 (13)	0.0319 (11)	0.0348 (12)	−0.0017 (10)	0.0174 (10)	−0.0035 (9)
C10	0.080 (2)	0.0624 (18)	0.0577 (18)	0.0262 (16)	0.0197 (16)	−0.0109 (14)
C12	0.0522 (14)	0.0317 (12)	0.0372 (12)	−0.0025 (10)	0.0112 (11)	−0.0012 (10)
C13	0.0681 (18)	0.0466 (15)	0.0426 (14)	0.0009 (13)	0.0192 (13)	−0.0045 (11)
C14	0.099 (2)	0.0566 (18)	0.0400 (15)	0.0049 (17)	0.0166 (16)	−0.0028 (13)
C15	0.084 (2)	0.065 (2)	0.0479 (17)	0.0118 (17)	−0.0044 (16)	−0.0003 (14)
C16	0.0635 (19)	0.072 (2)	0.0637 (19)	0.0156 (16)	0.0115 (15)	0.0039 (16)
C17	0.0572 (16)	0.0520 (16)	0.0481 (15)	0.0063 (13)	0.0166 (13)	0.0006 (12)
C20	0.0612 (19)	0.0661 (19)	0.079 (2)	0.0106 (14)	0.0405 (16)	0.0183 (15)

### Geometric parameters (Å, °)

**Table:**

Ni1—O2	2.0330 (15)	C8—C9	1.438 (3)
Ni1—O2^i^	2.0330 (15)	C9—N2	1.309 (3)
Ni1—O1	2.0361 (15)	C9—C10	1.497 (3)
Ni1—O1^i^	2.0361 (15)	N2—N1	1.399 (3)
Ni1—O3^i^	2.0651 (16)	N1—C11	1.374 (3)
Ni1—O3	2.0651 (16)	N1—C12	1.411 (3)
Cl1—C3	1.744 (3)	C10—H10A	0.9600
O1—C7	1.259 (3)	C10—H10B	0.9600
O2—C11	1.259 (3)	C10—H10C	0.9600
O3—C20	1.425 (3)	C12—C17	1.378 (3)
O3—H3A	0.8505	C12—C13	1.389 (3)
C1—C6	1.376 (3)	C13—C14	1.373 (4)
C1—C2	1.382 (4)	C13—H13	0.9300
C1—H1	0.9300	C14—C15	1.367 (4)
C2—C3	1.357 (4)	C14—H14	0.9300
C2—H2	0.9300	C15—C16	1.385 (4)
C3—C4	1.368 (4)	C15—H15	0.9300
C4—C5	1.377 (4)	C16—C17	1.376 (4)
C4—H4	0.9300	C16—H16	0.9300
C5—C6	1.376 (3)	C17—H17	0.9300
C5—H5	0.9300	C20—H20A	0.9600
C6—C7	1.494 (3)	C20—H20B	0.9600
C7—C8	1.416 (3)	C20—H20C	0.9600
C8—C11	1.429 (3)		
			
O2—Ni1—O2^i^	180.00 (10)	C7—C8—C9	132.1 (2)
O2—Ni1—O1	90.52 (6)	C11—C8—C9	105.41 (19)
O2^i^—Ni1—O1	89.48 (6)	N2—C9—C8	111.0 (2)
O2—Ni1—O1^i^	89.48 (6)	N2—C9—C10	118.2 (2)
O2^i^—Ni1—O1^i^	90.52 (6)	C8—C9—C10	130.6 (2)
O1—Ni1—O1^i^	180.00 (5)	C9—N2—N1	106.68 (18)
O2—Ni1—O3^i^	91.53 (7)	C11—N1—N2	111.53 (18)
O2^i^—Ni1—O3^i^	88.47 (7)	C11—N1—C12	128.86 (19)
O1—Ni1—O3^i^	90.39 (6)	N2—N1—C12	119.36 (18)
O1^i^—Ni1—O3^i^	89.61 (6)	O2—C11—N1	123.5 (2)
O2—Ni1—O3	88.47 (7)	O2—C11—C8	131.4 (2)
O2^i^—Ni1—O3	91.53 (7)	N1—C11—C8	105.20 (18)
O1—Ni1—O3	89.61 (6)	C9—C10—H10A	109.5
O1^i^—Ni1—O3	90.39 (6)	C9—C10—H10B	109.5
O3^i^—Ni1—O3	180.00 (6)	H10A—C10—H10B	109.5
C7—O1—Ni1	126.33 (14)	C9—C10—H10C	109.5
C11—O2—Ni1	116.87 (14)	H10A—C10—H10C	109.5
C20—O3—Ni1	120.58 (16)	H10B—C10—H10C	109.5
C20—O3—H3A	101.0	C17—C12—C13	120.3 (2)
Ni1—O3—H3A	116.0	C17—C12—N1	120.3 (2)
C6—C1—C2	120.9 (3)	C13—C12—N1	119.4 (2)
C6—C1—H1	119.6	C14—C13—C12	119.2 (3)
C2—C1—H1	119.6	C14—C13—H13	120.4
C3—C2—C1	119.5 (3)	C12—C13—H13	120.4
C3—C2—H2	120.2	C15—C14—C13	121.1 (3)
C1—C2—H2	120.2	C15—C14—H14	119.4
C2—C3—C4	120.9 (2)	C13—C14—H14	119.4
C2—C3—Cl1	120.0 (2)	C14—C15—C16	119.3 (3)
C4—C3—Cl1	119.0 (2)	C14—C15—H15	120.4
C3—C4—C5	119.1 (2)	C16—C15—H15	120.4
C3—C4—H4	120.4	C17—C16—C15	120.6 (3)
C5—C4—H4	120.4	C17—C16—H16	119.7
C6—C5—C4	121.3 (2)	C15—C16—H16	119.7
C6—C5—H5	119.4	C16—C17—C12	119.4 (3)
C4—C5—H5	119.4	C16—C17—H17	120.3
C5—C6—C1	118.2 (2)	C12—C17—H17	120.3
C5—C6—C7	121.1 (2)	O3—C20—H20A	109.5
C1—C6—C7	120.6 (2)	O3—C20—H20B	109.5
O1—C7—C8	122.9 (2)	H20A—C20—H20B	109.5
O1—C7—C6	116.40 (19)	O3—C20—H20C	109.5
C8—C7—C6	120.69 (19)	H20A—C20—H20C	109.5
C7—C8—C11	122.5 (2)	H20B—C20—H20C	109.5
			
O2—Ni1—O1—C7	−22.71 (18)	C6—C7—C8—C9	18.3 (4)
O2^i^—Ni1—O1—C7	157.29 (18)	C7—C8—C9—N2	−179.0 (2)
O3^i^—Ni1—O1—C7	−114.24 (18)	C11—C8—C9—N2	1.7 (3)
O3—Ni1—O1—C7	65.76 (18)	C7—C8—C9—C10	6.5 (5)
O1—Ni1—O2—C11	30.69 (16)	C11—C8—C9—C10	−172.8 (3)
O1^i^—Ni1—O2—C11	−149.31 (16)	C8—C9—N2—N1	0.8 (3)
O3^i^—Ni1—O2—C11	121.10 (16)	C10—C9—N2—N1	176.1 (2)
O3—Ni1—O2—C11	−58.90 (16)	C9—N2—N1—C11	−3.2 (3)
O2—Ni1—O3—C20	132.23 (18)	C9—N2—N1—C12	−177.8 (2)
O2^i^—Ni1—O3—C20	−47.77 (18)	Ni1—O2—C11—N1	155.00 (18)
O1—Ni1—O3—C20	41.70 (18)	Ni1—O2—C11—C8	−25.3 (3)
O1^i^—Ni1—O3—C20	−138.30 (18)	N2—N1—C11—O2	−176.1 (2)
C6—C1—C2—C3	1.6 (5)	C12—N1—C11—O2	−2.0 (4)
C1—C2—C3—C4	1.3 (5)	N2—N1—C11—C8	4.1 (3)
C1—C2—C3—Cl1	−179.2 (2)	C12—N1—C11—C8	178.1 (2)
C2—C3—C4—C5	−2.5 (5)	C7—C8—C11—O2	−2.6 (4)
Cl1—C3—C4—C5	178.1 (2)	C9—C8—C11—O2	176.8 (2)
C3—C4—C5—C6	0.7 (5)	C7—C8—C11—N1	177.1 (2)
C4—C5—C6—C1	2.1 (4)	C9—C8—C11—N1	−3.4 (3)
C4—C5—C6—C7	179.5 (3)	C11—N1—C12—C17	36.5 (4)
C2—C1—C6—C5	−3.3 (4)	N2—N1—C12—C17	−149.9 (2)
C2—C1—C6—C7	179.3 (3)	C11—N1—C12—C13	−145.0 (2)
Ni1—O1—C7—C8	3.6 (3)	N2—N1—C12—C13	28.7 (3)
Ni1—O1—C7—C6	−178.18 (14)	C17—C12—C13—C14	0.8 (4)
C5—C6—C7—O1	−126.5 (2)	N1—C12—C13—C14	−177.7 (2)
C1—C6—C7—O1	50.9 (3)	C12—C13—C14—C15	−0.4 (4)
C5—C6—C7—C8	51.8 (3)	C13—C14—C15—C16	−0.3 (5)
C1—C6—C7—C8	−130.8 (3)	C14—C15—C16—C17	0.6 (5)
O1—C7—C8—C11	15.7 (4)	C15—C16—C17—C12	−0.2 (5)
C6—C7—C8—C11	−162.5 (2)	C13—C12—C17—C16	−0.5 (4)
O1—C7—C8—C9	−163.6 (2)	N1—C12—C17—C16	178.0 (3)

Symmetry codes: (i) −*x*+1, −*y*, −*z*.

### Hydrogen-bond geometry (Å, °)

**Table:**

*D*—H···*A*	*D*—H	H···*A*	*D*···*A*	*D*—H···*A*
O3—H3A···N2^ii^	0.85	2.00	2.795 (2)	156

Symmetry codes: (ii) −*x*+1, *y*−1/2, −*z*+1/2.
